# The impact of remimazolam on postoperative neurocognitive disorder in elderly surgical patients: a systematic review and meta-analysis

**DOI:** 10.3389/fphar.2026.1871492

**Published:** 2026-08-24

**Authors:** Qiang Fu, Bhushan Sandeep, Yufang Yang, Hong Li, Li Xian, Jinlong Yuan, Xin Huang

**Affiliations:** 1 Department of Anesthesiology, The Third People’s Hospital of Chengdu, Chengdu, Sichuan, China; 2 Department of Cardio-Thoracic Surgery, West China School of Medicine, Sichuan University, Sichuan University Affiliated Chengdu Second People’s Hospital, Chengdu Second People’s Hospital, Chengdu, Sichuan, China; 3 Department of Anesthesiology, No. 363 Hospital, Chengdu, Sichuan, China

**Keywords:** postoperative cognitive dysfunction, postoperative delirium, postoperative neurocognitive disorder, propofol, remimazolam

## Abstract

**Background:**

Postoperative neurocognitive disorder (PND), including postoperative delirium (POD) and postoperative cognitive dysfunction (POCD), are prevalent and debilitating complications in elderly surgical patients. Remimazolam, a novel ultra-short-acting benzodiazepine with favorable hemodynamic and sedative properties, has not been fully evaluated for its impact on PND.

**Method:**

This systematic review and meta-analysis included studies retrieved from PubMed, Embase, Web of Science, and Cochrane library up to February 2026. Eligible studies compared remimazolam with other sedatives in elderly surgical patients and reported POD or POCD incidence. The primary outcome was the incidence of POD/POCD; secondary outcomes included postoperative MMSE scores, intraoperative opioids consumption, duration of post-anesthesia care unit (PACU) and hospital stay, and perioperative complications.

**Results:**

Fifteen randomized controlled trials (RCTs) involving 2,226 patients were analyzed. Remimazolam was not statistically associated with reduction in POD incidence on postoperative days 1 or 3, nor did it affect delirium subtypes or duration. However, remimazolam may be associated with a reduced risk of early POCD on days 1, 3, and 5. Remimazolam reduced intraoperative remifentanil consumption and the incidence of perioperative hemodynamic complications (hypotension and bradycardia). MMSE scores were higher on day 7, and no statistically significant differences were observed in postoperative nausea and vomiting, PACU stay, or hospital stay duration.

**Conclusion:**

Remimazolam was not associated with a reduction in POD incidence but may offer a protective effect against early POCD in elderly surgical patients. It provides a favorable hemodynamic profile, and its opioid-sparing properties indicate that it may be a viable sedative option in this population, though further well-designed trials are required.

**Systematic Review Registration:**

https://inplasy.com/inplasy-2026-4-0037/.

## 1 Introduction

Postoperative neurocognitive disorder (PND), encompassing postoperative delirium (POD), delayed neurocognitive recovery (DNR), and postoperative cognitive dysfunction (POCD), represents a spectrum of debilitating neurological complications following surgery and anesthesia that disproportionately affects older adults aged over 60 years (Evered et al., 2018; Bhushan et al., 2022; Fu et al., 2025; Lahiri et al., 2025; Liao et al., 2023). Operationally, these entities are distinguished by their temporal profiles and assessment methodologies. POD is diagnosed using validated instruments such as the Confusion Assessment Method (CAM), CAM-ICU, or 3D-CAM, and it manifests as an acute confusional state characterized by fluctuating attention, awareness, and cognition, occurring within the first 7 days postoperatively (Fu et al., 2025; Suga et al., 2025; Li et al., 2022). DNR describes subacute cognitive impairment that may persist during the first month after surgery; it is typically assessed through comprehensive neuropsychological test batteries and represents an intermediate stage of postoperative brain dysfunction (Evered et al., 2018; Lahiri et al., 2025). Meanwhile, POCD refers to persistent deficits in memory, executive function, and attention lasting beyond 30 days postoperatively and is typically identified through standardized neuropsychological testing or reliable change indices relative to baseline performance (Bhushan et al., 2021; Suraarunsumrit et al., 2024); it represents long-term cognitive dysfunction. Collectively, these conditions are independently associated with prolonged hospitalization, increased short- and long-term mortality, accelerated cognitive decline, and diminished quality of life, imposing a substantial burden on healthcare systems worldwide (Evered et al., 2018; Girard et al., 2018).

The pathophysiology of PND is multifactorial, involving neuroinflammation, cerebral hypoperfusion, oxidative stress, and neurotransmitter imbalance (Wang et al., 2025a; Subramaniyan and Terrando, 2019). Anesthetic agents have been increasingly recognized as potentially modifiable contributors to these mechanisms (Fu et al., 2025; Subramaniam et al., 2019; Li et al., 2025a; Ever et al., 2023). Current anesthetics practice relies on sedatives such as propofol, midazolam, and dexmedetomidine; however, their neuroprotective safety profiles remain contentious. Propofol demonstrates inconsistent neuroprotective effects (Suga et al., 2025; Sh et al., 2023), while midazolam may increase POD risk through enhanced central nervous system depression (Li et al., 2025b). Although dexmedetomidine exhibits anti-delirium properties, its utility is constrained by dose-dependent bradycardia and hypotension (Fu et al., 2025; Sh et al., 2023).

Benzodiazepines, including midazolam, have traditionally been associated with increased delirium risk in older adults due to their central nervous system depressant effects, accumulation of active metabolites, and prolonged elimination half-lives. This has led to benzodiazepine avoidance recommendation in delirium-prone populations (Aldecoa et al., 2024). Remimazolam, an ultra-short-acting benzodiazepine, has emerged as a promising anesthetic sedative with rapid onset and offset, predictable pharmacokinetics, minimal hemodynamic disturbance, and specific reversibility with flumazenil (Antonik et al., 2012; Wiltshire et al., 2012). Remimazolam, despite being a benzodiazepine, differs pharmacologically from traditional agents in several respects, but whether these differences translate to improved neurocognitive outcomes remains an open empirical question. Previous evidence indicates that remimazolam attenuates neuroinflammation and promotes microglial M2 polarization, potentially conferring neuroprotective effects (Wen et al., 2024; Mao et al., 2024; Zhao et al., 2024). Clinically, recent meta-analyses indicate that remimazolam does not increase POD incidence compared to non-benzodiazepine hypnotics and may improve early postoperative cognitive performance (Arias et al., 2025; Huang et al., 2025). Nevertheless, existing evidence remains heterogeneous, with variability in patient populations, anesthetic context, outcome definitions, and follow-up durations limiting definitive conclusions.

Furthermore, prior studies have several methodological limitations: many focused exclusively on delirium prevention without assessing POCD (Suga et al., 2025; Wang et al., 2025b; Li et al., 2025c); others restricted comparisons to remimazolam versus propofol alone, omitting dexmedetomidine and additional clinically relevant comparators (Suga et al., 2025); and few evaluated comprehensive safety endpoints including hemodynamic stability and opioid-sparing effects. Given these critical gaps in the evidence base, this systematic review and meta-analysis of randomized controlled trials (RCTs) was conducted to comprehensively evaluate the efficacy and safety of remimazolam on POD and POCD across multiple postoperative time-points. By stratifying outcomes temporally and phenotypically, and by including studies under both general and neuraxial anesthesia, this review provides clinically actionable insights to inform anesthetic decision-making in elderly surgical patients.

## 2 Methods

This systematic review’s protocol followed the Preferred Reporting Items for Systematic Reviews and Meta-Analyses (PRISMA) 2020 statement (Page et al., 2021) and was registered at International Platform of Registered Systematic Review and Meta-analysis Protocols (JVDS et al., 2023) (INPLASY202640037).

### 2.1 Data sources and search strategy

Two authors independently searched electronic databases, including PubMed, Embase, Web of science, and Cochrane Library databases, for published articles till 5 february 2026, to identify relevant studies. We used (“Postoperative Cognitive Complications,” OR “Postoperative cognitive dysfunction,” OR “Postoperative delirium,” OR “Postoperative neurocognitive disorder,” OR “Perioperative neurocognitive disorder”) AND (“Remimazolam”) for the subject and free word searches. The detailed search strategy is provided in the supplemental digital content. References deemed relevant by any reviewer had their full text retrieved; two independent reviewers determined eligibility. Restrictions on language and publication date were not applied. We documented the most pertinent reason for excluding articles after a full-text review. Discrepancies were resolved through discussion.

### 2.2 Eligibility criteria

RCTs meeting the following PICOS criteria were included: (P) Population: adult patients (aged ≥18 years) undergoing elective surgery under general or neuraxial anesthesia, regardless of sex or nationality; (I) intervention: remimazolam was used as the sedative for induction or maintenance; (C) comparator: active comparators (propofol, dexmedetomidine, or midazolam) or placebo (normal saline), with balanced use of concomitant anesthetic agents (e.g., inhalational anesthetics and opioids) between groups; (O) outcomes: the outcome included the incidence of PND, POD, or POCD. The exclusion criteria included the following: (i) studies conducted exclusively in ICU settings were excluded, as ICU delirium involves etiological factors distinct from postoperative delirium; (ii) emergency surgery, given uncontrolled confounders including hemodynamic instability and incomplete preoperative assessment; (iii) studies involving individuals with pre-existing brain diseases; (iv) cardiac surgery with cardiopulmonary bypass, which independently induces neurocognitive dysfunction through systemic inflammatory and microembolization; (v) intracranial neurosurgery, given the direct operative effects on brain parenchyma independent of exposure to anesthetics; and (vi) procedural sedation without surgical intervention (e.g., endoscopy and bronchoscopy).

Although the formal eligibility criteria specified adult patients aged ≥18 years to ensure comprehensive literature capture, the incidence of PND increases in elderly populations (Duran et al., 2024). Consequently, this review effectively evaluates the neurocognitive effects in older surgical patients, which is a clinically appropriate focus given that (i) PND incidence increases exponentially after age 60 and (ii) the favorable hemodynamic profiles of remimazolam is particularly relevant for elderly patients with diminished cardiovascular reserve.

### 2.3 Primary and secondary outcomes

The primary outcome was the incidence of PND, POD, or POCD. Studies that utilized valid tools for diagnosing delirium, such as the CAM, Mini-Mental State Examination (MMSE), Richmond Agitation-Sedation Scale (RASS), Nursing Delirium Screening Scale (Nu-DESC scoring), and Montreal Cognitive Assessment (MoCA), were included in our review. The secondary outcomes included postoperative MMSE scores at different time-points, consumption of intraoperative opioids (remifentanil and sufentanil), duration of post-anesthesia care unit (PACU) and hospital stay, and perioperative complications [postoperative nausea and vomiting (PONV), post-induction hypotension, intraoperative bradycardia, postoperative respiratory depression, and intra-operative awareness].

### 2.4 Data extraction

The following data were extracted from individual studies: 1) first author and publication year; 2) country; 3) number of patients in each study; 4) types of surgery; 5) American Society of Anesthesiologists Physical Status Classification; 6) specific interventions and comparisons, including the name and dose of the drugs and the method of administration; 7) intraoperative sedation depth monitoring; 8) Clinic registration number.

### 2.5 Risk of bias and evidence quality assessment

Evaluation with Cochrane risk-of-bias tool was independently completed by two researchers, with any disagreements resolved via discussion (Sterne et al., 2019). We assessed the certainty of the evidence for each outcome using the Grading of Recommendations, Assessment, Development, and Evaluation (GRADE) framework (Atkins et al., 2004), which assesses the following domains: 1) risk of bias of included studies; 2) indirectness, inconsistency, and imprecision of the effect estimates; and 3) additional considerations, including the risk of publication bias. For each outcome, the certainty of the evidence was classified as ‘high,’ ‘moderate,’ ‘low,’ or ‘very low.’

### 2.6 Data synthesis

Data were analyzed using the random-effects model in RevMan 5.4.1 (The Cochrane Collaboration). Heterogeneity was assessed using the I^2^ statistic. Given the a priori clinical heterogeneity across the included studies in the surgical type, comparator agents, anesthesia techniques, and outcomes assessment methods, random-effects models were used as the primary analysis for all outcomes. Fixed-effects models were applied as sensitivity analyses (Chen and Benedetti, 2017; Bowden et al., 2011). For dichotomous variables, we extracted the number of occurrences and the total number of the sample. Continuous data were expressed as means and standard deviations (SD), and standard mean differences (SMD) with 95% confidence intervals (CIs) were calculated. For studies reporting medians and interquartile ranges (IQR), these values were converted to means and SD using the method described by Hozo et al. (2005). Dichotomous data were analyzed as relative risks (RRs) with 95% CIs using the Mantel–Haenszel method. Additionally, subgroup analysis was conducted among studies that assessed for POD based on different types of surgery and anesthetics. Sensitivity analyses were conducted using the leave-one-out approach to identify potential sources of heterogeneity for the primary outcome.

To avoid redundant sample size assessments in multi-arm trials, participants were evenly distributed across groups. In designs with one intervention group and two control groups, patients in the intervention group were proportionally allocated to allow pairwise comparisons with each control group. Funnel plots were generated to evaluate potential publication bias and small-study effects for outcomes with data from ≥10 included studies. P < 0.05 was considered statistically significant.

## 3 Results

### 3.1 Study selection

**FIGURE 1:**
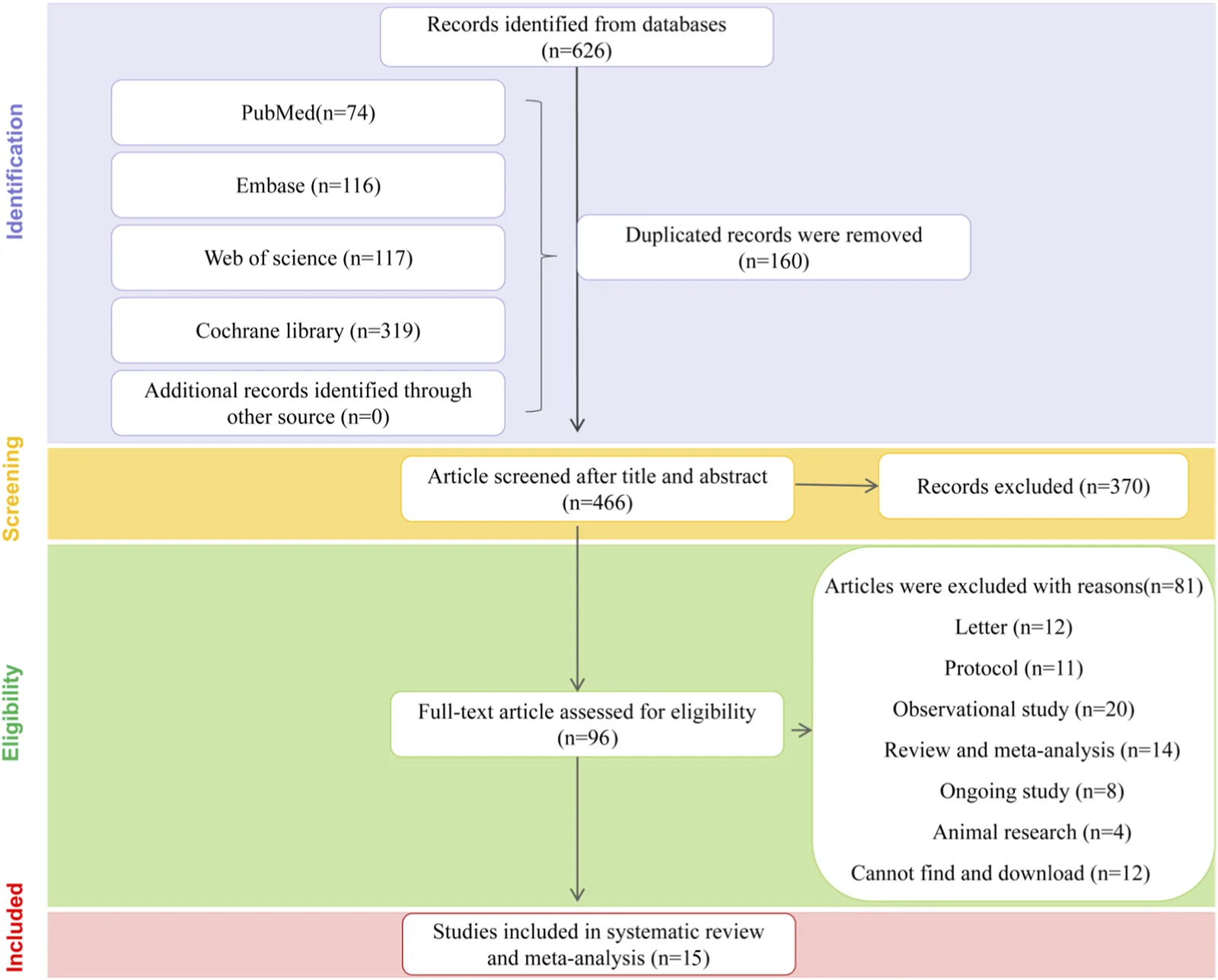
PRISMA flow diagram of the included and excluded studies. PRISMA, Preferred Reporting Items for Systematic Reviews and Meta-Analyses.

**FIGURE 2:**
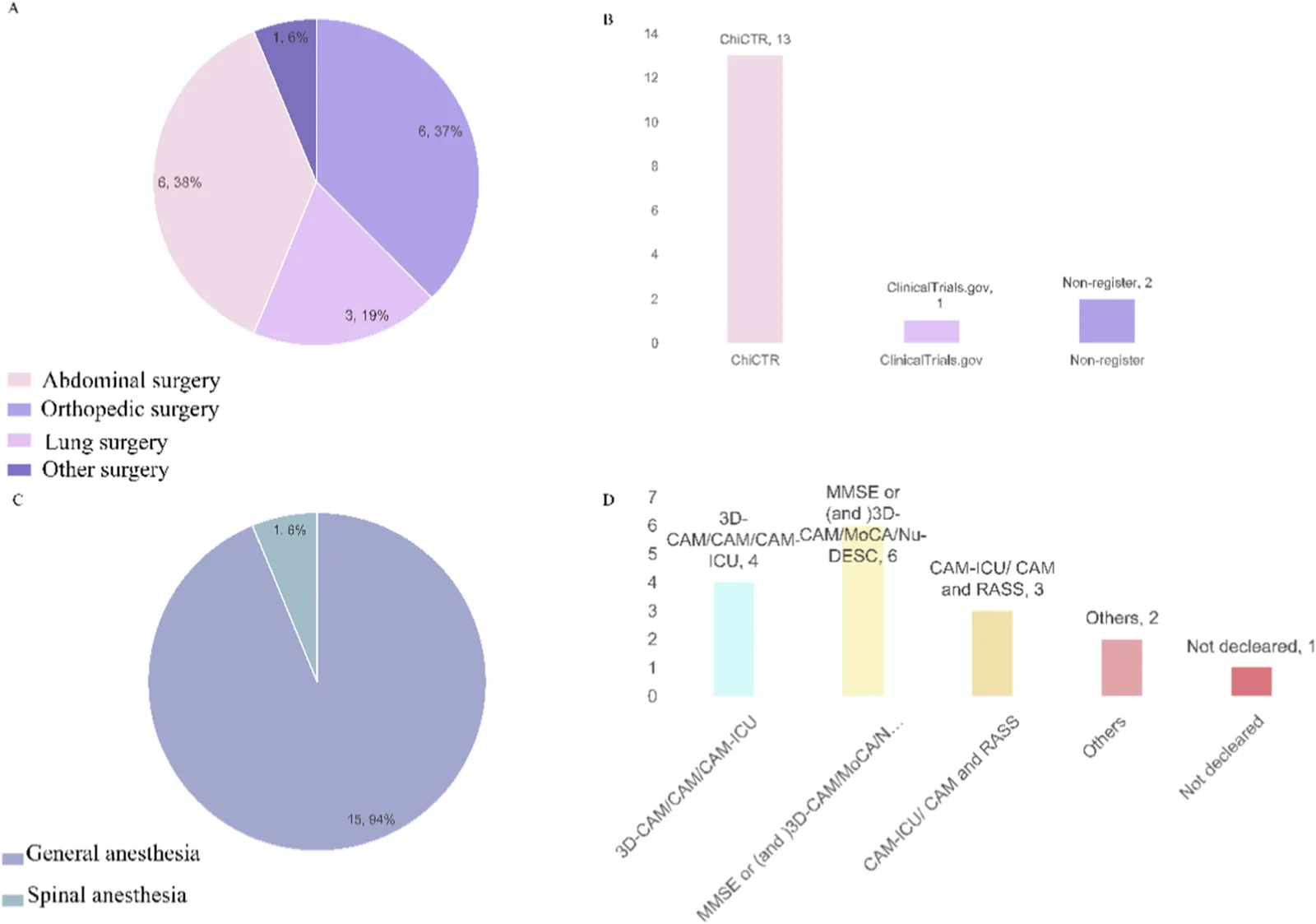
Characteristics of the included studies, including the surgery type **(A)**, trial registration **(B)**, anesthesia methods **(C)**, and cognitive assessment tools **(D)**.

The search strategy retrieved 626 records, 96 of which were eligible for full-text review after screening the title and abstract. We excluded 81 studies analyzing different populations, outcomes, and settings according to the predefined eligibility protocol. Fifteen RCTs, with 2,226 participants, met the eligibility criteria (Figure 1) (Liao et al., 2023; Cai et al., 2025; Chen et al., 2025; Duan et al., 2024; Gao et al., 2023; Hu et al., 2025; Kuang et al., 2023; Liu et al., 2025a; Liu et al., 2025b; Qiao et al., 2025; Liu et al., 2024; Wang et al., 2025c; Wu et al., 2024; Yang et al., 2023; Yaqiu et al., 2025; Zhang et al., 2026). The key characteristics of all 15 included trials are summarized in Table 1. The mean age ranged from 65.2 to 77.5 years. All patients underwent elective procedures, including orthopedic surgery (n = 6) (Cai et al., 2025; Duan et al., 2024; Wang et al., 2025c; Wu et al., 2024; Yang et al., 2023; Yaqiu et al., 2025), lung surgery (n = 3) (Chen et al., 2025; Hu et al., 2025; Kuang et al., 2023), major abdominal surgery (n = 5) (Liao et al., 2023; Liu et al., 2025a; Liu et al., 2025b; Liu et al., 2024; Wang et al., 2025c; Zhang et al., 2026), and other surgeries (n = 1)^46^ under general anesthesia (Liao et al., 2023; Cai et al., 2025; Chen et al., 2025; Duan et al., 2024; Hu et al., 2025; Kuang et al., 2023; Liu et al., 2025a; Liu et al., 2025b; Liu et al., 2024; Wang et al., 2025c; Wu et al., 2024; Yang et al., 2023; Yaqiu et al., 2025; Zhang et al., 2026) or spinal anesthesia (Qiao et al., 2025; Wang et al., 2025c). Thirteen studies used a two-arm design (Cai et al., 2025; Chen et al., 2025; Duan et al., 2024; Hu et al., 2025; Kuang et al., 2023; Liu et al., 2025a; Qiao et al., 2025; Liu et al., 2024; Wang et al., 2025c; Wu et al., 2024; Yang et al., 2023; Yaqiu et al., 2025; Zhang et al., 2026), one used a three-arm design (Liao et al., 2023), and one used a four-arm design (Liu et al., 2025b). To prevent unit-of-analysis errors arising from repeated inclusion of the shared remimazolam arm in multi-arm trials (Liao et al., 2023; Liu et al., 2025b), we divided the remimazolam group into two equal halves for separate pairwise comparisons with each control group. Figures 2A–D show the characteristics of the included studies, including the surgery type, trial registration, anesthesia methods, and cognitive assessment tools.

**TABLE 1:** Characteristics of the included studies. ASA: American Society of Anesthesiologists; DEX: dexmedetomidine; VATS: video-assisted thoracoscopic surgery; TURP, transurethral resection of the prostate; TKA, total knee arthroplasty; PKR, partial knee replacement. Data are presented as the mean ± SD, median [IQR], or n (%).

Study	Country	Mean age	No of patients	Surgery type	Asa	Intervention (remimazolam)	Comparison	Intraoperative sedation depth monitoring	Trial registration
Cai et al., 2025	China	75 (71–80.8) vs. 77.5 (73–82.8)	68 vs. 68	Hip surgery	II–III	Induction of anesthesia: 0.2 mg/kg–0.3 mg/kg Maintenance: 0.3 mg/kg/h–1.0 mg/kg/h	Propofol induction: 1.0 mg/kg–1.5 mg/kg Maintenance: 4 mg/kg/h–12 mg/kg/h	SedLine brain function monitoring (patient state index, PSI)	ChiCTR2300068632
Chen et al., 2025	China	69 (67–71) vs. 68 (67–72)	88 vs. 88	VATS	I–III	0.5 mg/kg/h starting 3 min before anesthesia induction until surgery completion	DEX: 0.5 μg/kg/h starting 3 min before anesthesia induction until surgery completion	Bispectral index	ChiCTR2500101977
Duan et al., 2024	China	77.4 ± 6.1 vs. 75.3 ± 7.7	53 vs. 53	Hip fracture surgery	II–III	Maintained by remimazolam at 0.1 mg/kg/h–0.3 mg/kg/h after a loading dose of 0.05 mg/kg	Propofol: 0.3 mg/kg–0.5 mg/kg, followed by a pump infusion at 0.5 mg/kg/h–3 mg/kg/h for maintenance	Bispectral index	ChiCTR2200067024
Hu et al., 2025	China	69 (65–72) vs. 67 (62–71)	46 vs. 46	VATS	II–III	Induction: 0.25 mg/kg; maintenance: 0.5 mg/kg/h–1.5 mg/kg/h	Propofol induction: 2 mg/kg; maintenance: 4 mg/kg/h–6 mg/kg/h	Bispectral index	ChiCTR2400094127
Kuang et al., 2023	China	65.4 ± 3.9 vs. 65.2 ± 4.4	42 vs. 42	Pulmonary lobectomy	I–III	Induction: 0.3 mg/kg; maintained with 0.6 mg/kg/h–1.2 mg/kg/h	Propofol induction: 2.0 mg/kg; maintained with propofol (2–10) mg/kg/h	Bispectral index	ChiCTR2100050236
Liao et al., 2023	China	70.12 ± 3.57 vs. 71.26 ± 3.58 vs. 69.69 ± 2.52	34 vs. 35 vs. 35	Laparoscopic radical resection of gastric cancer	II–III	Induction: 0.2 mg/kg; maintained with 0.3 mg/kg/h–0.5 mg/kg/h	1. DEX induction: 0.5 μg/kg; maintained with 0.3 mg/kg/h–0.5 μg/kg/h 2. Saline 5 mL, maintained with saline 0.3 mg/kg/h–0.5 mL/kg/h	Bispectral index	ChiCTR2300068036
Liu et al., 2025a	China	68.25 ± 5.46 vs. 69.45 ± 5.89	56 vs. 56	Laparoscopic cholecystectomy	I–III	Induction: 0.2 mg/kg; maintained with 0.5 mg/kg/h–1.0 mg/kg/h	Propofol induction: 1.5 mg/kg; maintained with 4 mg/kg/h–8 mg/kg/h	Bispectral index	ChiCTR2300077536
Liu et al., 2024	China	71.620 ± 5.473 vs. 71.400 ± 5.500	50 vs. 50	Radical resection of colon cancer	I–III	Induction: 0.1 mg/kg–0.2 mg/kg; maintained with 0.4 mg/kg/h–1.2 mg/kg/h	Propofol induction: 1 mg/kg–2 mg/kg; maintained with 4 mg/kg/h–10 mg/kg/h	Bispectral index	ChiCTR2300076384
Liu et al., 2025b	China	73.9 ± 6.3 vs. 69.7 ± 4.4 vs. 72.3 ± 5.6 vs. 71.5 ± 5.2	30 vs. 28 vs. 30 vs. 29	Laparoscopic colorectal cancer surgery	II–III	R1: 0.5 mg/kg/h R2: 1.0 mg/kg/h R3: 1.5 mg/kg/h	Propofol maintained with propofol at 1 mg/kg/h–10 mg/kg/h	Bispectral index	ChiCTR2400083122
Qiao et al., 2025 (multi-center, neuraxial anesthesia)	China	69.9 ± 4.7 vs. 69.9 ± 4.5	160 vs. 78	Cystoscopy, TURP, hemorrhoidectomy, surgical amputation, TKA, and PKR	I–III	Induction: 5.0 mg; maintained with 0.2 mg/kg/h	Saline induction: 5 mL of 0.9% saline; maintained with 0.2 mL/kg/h	Bispectral index	ChiCTR2100048744
Wang et al., 2025a (Spinal anesthesia)	China	71 (67–77) vs. 71 (66–78)	63 vs. 63	Lower extremity orthopedic surgery	I–III	Induction: 0.1 mg/kg; maintained with 0.1 mg/kg/h–0.3 mg/kg/h	DEX induction: 0.5 μg/kg; maintained with 0.2 mg/kg/h–0.6 μg/kg/h	Modified Observer’s assessment of Alertness/Sedation	ChiCTR2400083380
Wu et al., 2024	China	72.23 ± 4.39 vs. 71.97 ± 4.88	35 vs. 35	Lower extremity fractures surgeries	I–III	Induction: 0.1 mg/kg; maintained with 0.1 mg/kg/h	Midazolam induction: 0.05 mg/kg; maintained with 0.1 mL/kg/h	Electrocardiograph	ClinicalTrials.gov website (NCT04601350)
Yang et al., 2023	China	68 (65–71) vs. 68 (65–71)	147 vs. 153	Orthopedic surgery	I–III	Induction: 0.2 mg/kg–0.3 mg/kg; maintained with intravenous infusion of remimazolam	Propofol induction: 1.0 mg/kg–1.5 mg/kg; maintained with intravenous infusion of propofol	Bispectral index	ChiCTR2100050372
Li et al., 2025a	China	73.96 ± 8.02 vs. 72.31 ± 7.13	60 vs. 60	Spinal surgery	II–III	Induction: 0.2 mg/kg–0.3 mg/kg; maintained with 0.1 mg/kg/h–0.2 mg/kg/h	Propofol induction: 1.5 mg/kg–2.0 mg/kg; maintained with 4 mg/kg–6 mg/kg/h	Bispectral index	-
Zhang et al., 2026	China	71 (69–75) vs. 70 (69–73)	173 vs. 170	Major abdominal surgery	II–III	Induction: 0.1 mg/kg–0.3 mg/kg; maintained with intravenous infusion of remimazolam	Propofol induction: 1 mg/kg–3 mg/kg; maintained with intravenous infusion of propofol	Bispectral index	-

**TABLE 2:** Perioperative cognitive assessment and pain management. POD, postoperative delirium; 3D-CAM, 3-minute diagnostic confusion assessment method; PCA, patient-controlled analgesia; CAM: confusion assessment method; TPVB: thoracic paravertebral block; MMSE: mini-mental state examination; PCIA: patient-controlled intravenous analgesia; POCD: postoperative cognitive dysfunction; VFT: verbal fluency test; CDT: clock drawing test; DSST: digit symbol substitution test; AVLT-H: auditory verbal learning test–Huashan; CAM-ICU, confusion assessment method for the intensive care unit scale; RASS, Richmond agitation–sedation scale; MoCA: Montreal cognitive assessment.

Study	Postoperative cognitive assessment period	Cognitive assessment tools	Multimodal analgesia
Cai et al., 2025	Incidence of POD within three postoperative days	3D-CAM	PCA
Chen et al., 2025	Incidence of POD within five days after surgery	CAM	1. TPVB 2. Flurbiprofen axetil 50 mg
Duan et al., 2024	Incidence of POD within seven postoperative days	CAM	PCIA
Hu et al., 2025	Incidence of PND on postoperative days 1, 3, 5, and 7	3D-CAM and MMSE	PCIA
Kuang et al., 2023	Cognitive function was assessed with neuropsychological tests one day before surgery and seven days after surgery	MMSE, VFT, CDT, DSST, and AVLT-H	-
Liao et al., 2023	One day before the procedure, three days after the procedure, and seven days after the procedure	MMSE and MoCA	1. TAPB combined with rectus abdominis sheath blocks 2. PCA
Liu et al., 2025a	1. Preoperatively, postoperative day 3, and postoperative day 7 2. Postoperative days 1, 3, and 7	1.MMSE and MoCA for POCD 2.CAM for POD	-
Liu et al., 2024	Incidence of POD within seven postoperative days	CAM-ICU and RASS	1. PCA 2. 50 mg flurbiprofen
Liu et al., 2025b	Preoperatively, and on postoperative days 3 and 7	MMSE and MoCA	PCIA
Qiao et al., 2025 (multi-center)	Preoperatively and within seven days	Nu-DESC, MMSE, and MoCA	-
Wang et al., 2025a	Postoperatively	-	Postoperative regional blocks
Wu et al., 2024	Cognitive function was assessed preoperatively and on postoperative days 1, 3, 5, and 7	MMSE	-
Yang et al., 2023	Incidence of delirium within three days after surgery	CAM and RASS	PCIA
Li et al., 2025a	Occurrence of POD was recorded from one to seven days postoperatively	State of consciousness assessment score	1. PCA 2. 50 mg tramadol
Zhang., et al., 2026	Incidence of delirium within five days postoperatively	CAM or CAM-ICU	PCIA

### 3.2 Primary outcome

#### 3.2.1 Incidence of POD

**FIGURE 3:**
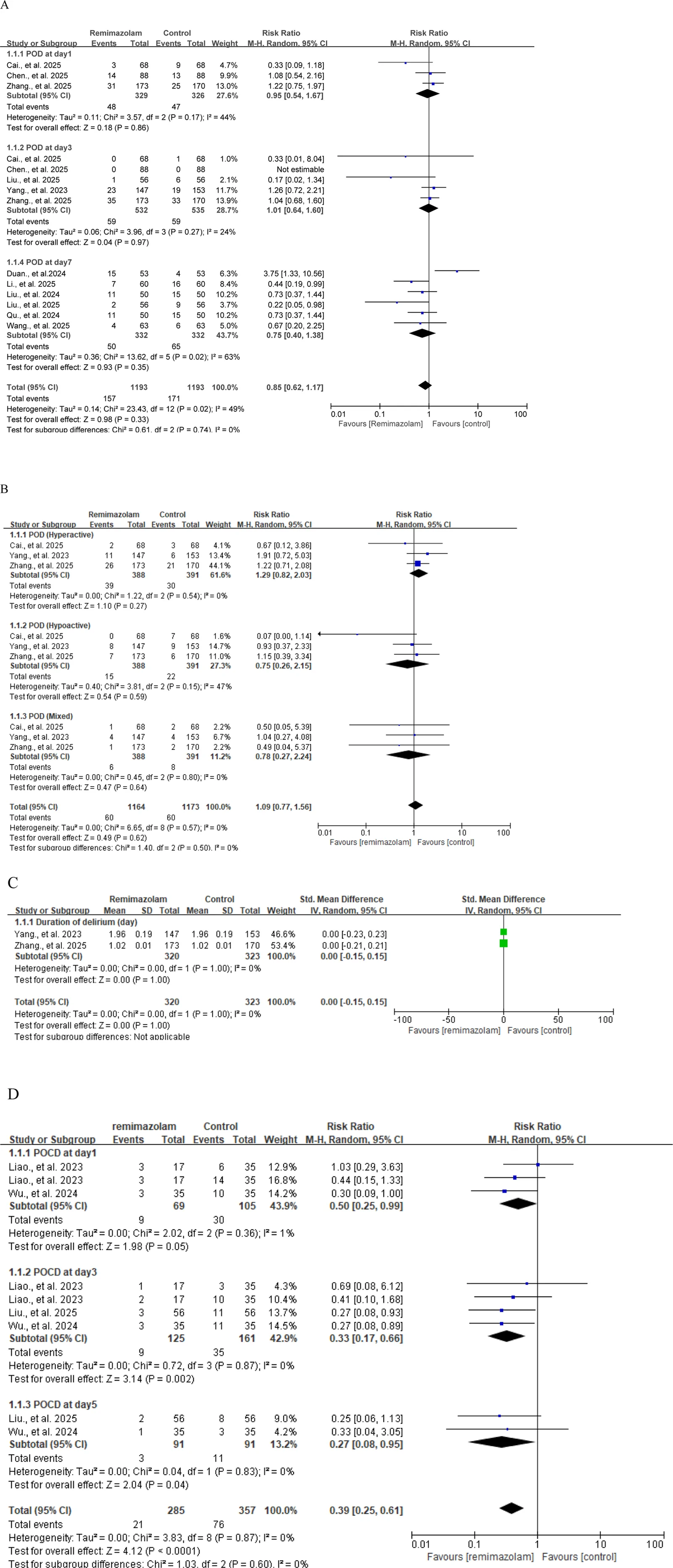
Forest plot of the incidence of POD and POCD between remimazolam and control. **(A)** Forest plot of the incidence of POD. **(B)** Forest plot of the subtype of delirium. **(C)** Forest plot of the duration of delirium. **(D)** Forest plot of the incidence of POCD. POD: postoperative delirium; POCD: postoperative cognitive dysfunction.

No statistically significant difference was observed between the remimazolam and control groups on postoperative day 1 [RR, 0.92; 95% CI, 0.54 to 1.67; I^2^, 44%; P, 0.86] (Cai et al., 2025; Chen et al., 2025; Zhang et al., 2026), day 3 [RR, 1.01; 95% CI, 0.64 to 1.60; I^2^, 24%; P, 0.97] (Cai et al., 2025; Chen et al., 2025; Liu et al., 2025a; Yang et al., 2023; Zhang et al., 2026), But reduced incidence of POD at day7 [RR, 0.61; 95 % CI, 0.39 to 0.93; I^2^=0 %; P=0.02] (Duan et al., 2024; Liu et al., 2025a; Liu et al., 2024; Wang et al., 2025c; Yaqiu et al., 2025) in terms of POD (Figure 3A). Three studies differentiated the types of delirium (Cai et al., 2025; Yang et al., 2023; Zhang et al., 2026) and revealed that there is no statistically significant difference observed in hyperactive [RR, 1.29; 95% CI, 0.82 to 2.03; I^2^, 0%; P, 0.27] (Cai et al., 2025; Yang et al., 2023; Zhang et al., 2026), hypoactive [RR, 0.75; 95% CI, 0.26 to 2.15; I^2^, 47%; P, 0.59] (Cai et al., 2025; Yaqiu et al., 2025; Zhang et al., 2026), or mixed [RR, 1.09; 95% CI, 0.27 to 2.24; I^2^, 0%; P, 0.64] (Cai et al., 2025; Yang et al., 2023; Zhang et al., 2026) delirium (Figure 3B). Subgroup analysis based on different surgeries also revealed that the incidence of POD shows no statistically significant difference on day 3 [RR, 1.21; 95% CI, 0.69 to 2.11; I^2^, 0%; P, 0.50] (Cai et al., 2025; Yang et al., 2023) or day 7 [RR, 1.02; 95% CI, 0.26 to 3.92; I^2^, 81%; P, 0.98] (Duan et al., 2024; Wang et al., 2025c; Yaqiu et al., 2025) postoperatively (Supplementary Figure 1). Moreover, only one study assessed the onset time of delirium (Yang et al., 2023), and two studies that recorded the duration of delirium revealed no statistically significant difference between the remimazolam and control groups [SMD, 0.00; 95% CI, −0.15 to 0.15; I^2^, 0%; P, 1.00] (Yang et al., 2023; Zhang et al., 2026) (Figure 3C).

Similarly, no statistically significant difference were observed between remimazolam and propofol maintenance anesthesia at day1 [RR, 0.73; 95% CI, 0.21 to 2.55; I^2^ = 72%; P = 0.62], day2 [RR, 0.91; 95% CI, 0.38 to 2.22; I^2^ = 13%; P = 0.84], day3 [RR, 1.01; 95% CI, 0.64 to 1.60; I^2^ = 24%; P = 0.97], or day7 [RR, 0.76; 95 % CI, 0.28 to 2.06; I^2^=78 %; P=0.58] (Supplementary Figure 2). Moreover, sensitivity analysis, conducted by sequentially removing studies one by one, identified the source of heterogeneity, which remained low (Supplementary Table S1).

#### 3.2.2 Incidence of POCD

We found a statistically significant difference in the risk of POCD in the control group compared with that in the remimazolam group on day 1 [RR, 0.50; 95% CI, 0.25 to 0.99; I^2^, 1%; P, 0.05] (Liao et al., 2023; Wu et al., 2024), day 3 [RR, 0.33; 95% CI, 0.17 to 0.66; I^2^, 0%; P, 0.002] (Liao et al., 2023; Liu et al., 2025a; Wu et al., 2024), and day 5 [RR, 0.27; 95% CI, 0.08 to 0.95; I^2^, 0%; P, 0.04] (Liu et al., 2025a; Wu et al., 2024) (Figure 3D). Moreover, sensitivity analysis, conducted by sequentially removing the studies one by one, identified the source of heterogeneity, which remained low-to-moderate (Supplementary Table S1).

### 3.3 Secondary outcomes

#### 3.3.1 Postoperative MMSE scores at different time-points

**FIGURE 4:**
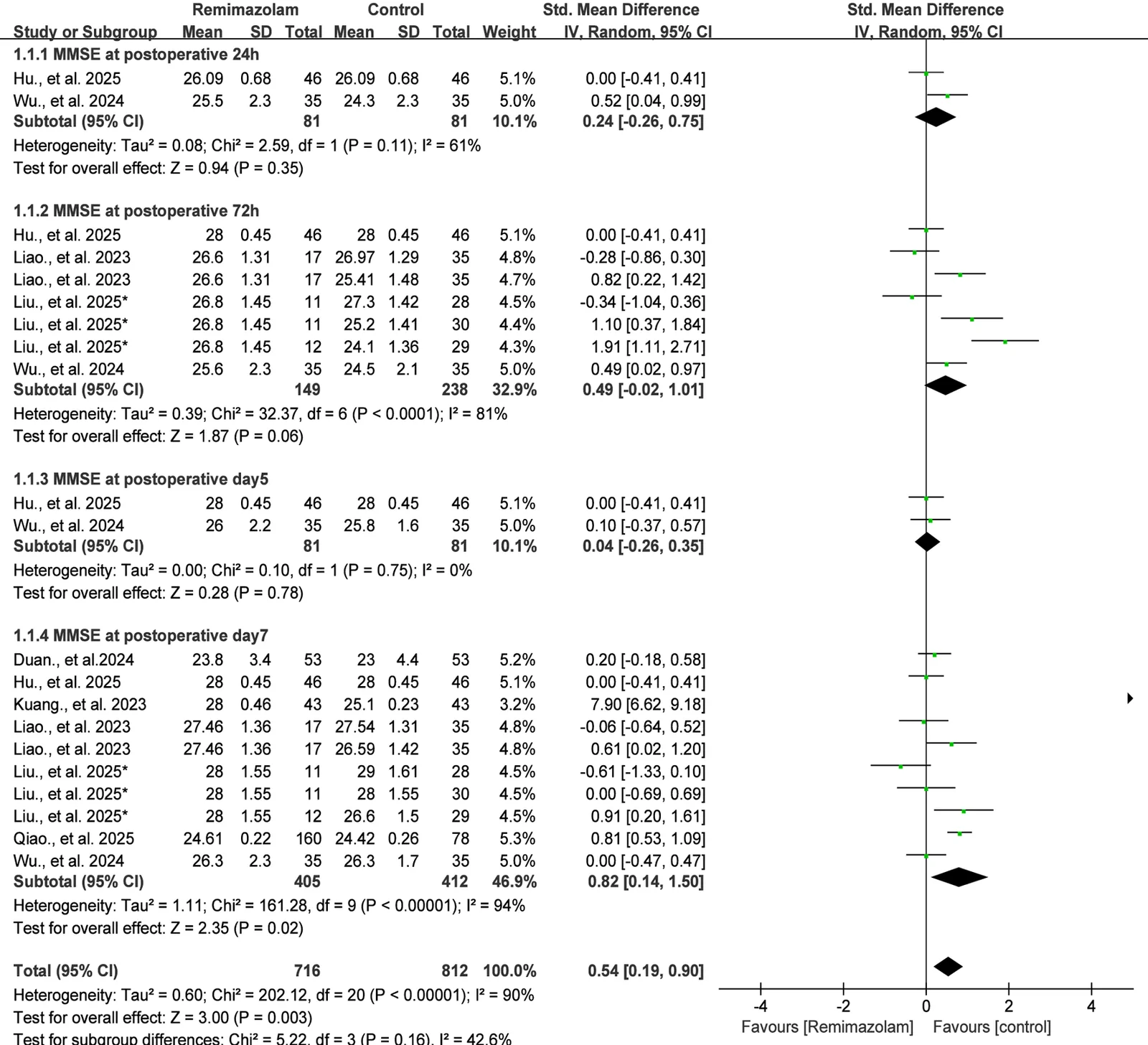
Forest plot of the postoperative MMSE scores at different time-points between remimazolam and control. MMSE: mini-mental state examination.

No statistically significant difference was observed in the postoperative MMSE scores at 24 h [SMD, 0.24; 95% CI, −0.26 to 0.75; I^2^, 61%; P, 0.35] (Hu et al., 2025; Wu et al., 2024), 72 h [SMD, 0.49; 95% CI, −0.02 to 1.01; I^2^, 81%; P, 0.06] (Liao et al., 2023; Hu et al., 2025; Liu et al., 2025b; Wu et al., 2024), and on day 5 [SMD, 0.04; 95% CI, −0.26 to 0.35; I^2^, 0%; P, 0.78] (Hu et al., 2025; Wu et al., 2024). Nevertheless, MMSE scores on day 7 appeared higher in the remimazolam group than in the control group [SMD, 0.82; 95% CI, 0.14 to 1.50; I^2^, 94%; P, 0.02] (Liao et al., 2023; Duan et al., 2024; Hu et al., 2025; Kuang et al., 2023; Liu et al., 2025b; Qiao et al., 2025; Wu et al., 2024) (Figure 4). However, this pooled estimate is characterized by extreme statistical heterogeneity (I^2^, 94%; 95% CI, 89%–97%), indicating that the true effect varies substantially across studies.

#### 3.3.2 Consumption of intraoperative opioids

Remimazolam has significantly reduced the amount of remifentanil consumption compared with control group [SMD, -0.56; 95 % CI, -1.01 to -0.11; I^2^=88%; P=0.01] (Liao et al., 2023; Cai et al., 2025; Chen et al., 2025; Hu et al., 2025; Liu et al., 2025b; Liu et al., 2024; Wang et al., 2025c; Wu et al., 2024). However, we found no statistically significant difference in sufentanil consumption between the two groups [SMD, −0.70; 95% CI, −1.72 to 0.33; I^2^, 96%; P, 0.18] (Chen et al., 2025; Duan et al., 2024; Hu et al., 2025; Wu et al., 2024) (Supplementary Figure 3). Liu et al., 2025b

#### 3.3.3 Duration of PACU and hospital stay

No statistically significant differences were observed in the duration of PACU [SMD, −0.31; 95% CI, −1.05 to 0.43; I^2^, 97%; P, 0.41] (Liao et al., 2023; Cai et al., 2025; Hu et al., 2025; Liu et al., 2025a; Liu et al., 2025b; Wu et al., 2024; Yang et al., 2023; Yaqiu et al., 2025) and hospital stay [SMD, 0.68; 95% CI, −0.10 to 1.45; I^2^, 97%; P, 0.09] (Cai et al., 2025; Chen et al., 2025; Duan et al., 2024; Yang et al., 2023; Zhang et al., 2026) between the remimazolam and control groups (Supplementary Figure 4).

#### 3.3.4 Perioperative complications

Eleven studies that analyzed PONV disclosed there is no statistically significant difference between the remimazolam and control groups [RR, 1.07; 95 % CI, 0.82 to 1.38; I^2^=0%; P=0.63] (Liao et al., 2023; Cai et al., 2025; Chen et al., 2025; Duan et al., 2024; Hu et al., 2025; Liu et al., 2025a; Liu et al., 2025b; Liu et al., 2024; Wang et al., 2025c; Yang et al., 2023). However, remimazolam reduced the post-induction hypotension [RR, 0.38; 95% CI, 0.30 to 0.49; I^2^, 14%; P < 0.00001] (Liao et al., 2023; Duan et al., 2024; Hu et al., 2025; Kuang et al., 2023; Liu et al., 2025a; Wang et al., 2025c; Wu et al., 2024; Yang et al., 2023; Yaqiu et al., 2025) and intraoperative bradycardia [RR, 0.35; 95% CI, 0.19 to 0.61; I^2^, 60%; P, 0.0003] (Liao et al., 2023; Duan et al., 2024; Hu et al., 2025; Kuang et al., 2023; Liu et al., 2025a; Wang et al., 2025c; Wu et al., 2024) compared with that in the control group. However, no statistically significant difference was observed in postoperative respiratory depression [RR, 0.55; 95 % CI, 0.21 to 1.44; I^2^=12%; P=0.22] (Liao et al., 2023; Hu et al., 2025; Liu et al., 2025b; Liu et al., 2024; Wang et al., 2025c; Wu et al., 2024; Yaqiu et al., 2025) compared with that in the control group (Supplementary Figure 5). Furthermore, two studies revealed that no statistically significant difference in intra-operative awareness was found between the remimazolam and control groups (Cai et al., 2025; Yang et al., 2023).

### 3.4 Risk of bias assessment

All included RCTs were judged to be at low risk of bias across the five quality domains (Supplementary Table S2). Regarding the randomization process, two studies were rated as having “some concerns” due to unclear allocation concealment methods (Duan et al., 2024; Yaqiu et al., 2025). For deviations from intended intervention, all 15 studies were judged as low risk; most studies used standardized anesthesia protocols with specified drug dosing and titration targets. Missing outcome data were rated as low risk across all studies. Regarding the measurement of the outcome, 14 studies were rated as low risk; one study (Yaqiu et al., 2025) raised some concerns because cognitive assessment was performed by unblinded research staff, though the consciousness assessment scores were objective. Selection of the reported result was rated as low risk for all studies.

The certainty of evidence was rated using the Cochrane GRADE approach, with the initial high certainty for RCT-based outcomes subsequently downgraded for the risk of bias, inconsistency, indirectness, and imprecision, as appropriate. Notably, several outcomes rated as moderate or low certainty reflected imprecision due to wide confidence intervals spanning both null and clinically important effects and indirectness arising from heterogeneous cognitive assessment tools and outcome definition across trials. We acknowledge that the evidence base has important limitations, and our conservative downgrading approach reflects these uncertainties (Supplementary Table S3).

## 4 Discussion

The current systematic review and meta-analysis comprehensively evaluated the protective effects of remimazolam on PND in adult surgical patients. The primary findings revealed that remimazolam does not reduce the incidence of POD at any time-point up to 3 days postoperatively, nor does it affect delirium subtypes or duration. However, remimazolam may be associated with a reduced risk of POD on days 7 and, POCD on days 1, 3 and 5, while demonstrating a favorable hemodynamic profile and reduced intraoperative remifentanil consumption compared with control sedatives (propofol, dexmedetomidine, midazolam, or saline). These results may highlight a divergent effect of remimazolam on acute POD subtypes, with potential neuroprotective effects against early POCD, alongside safety advantages in perioperative care.

The absence of a statistically significant difference in POD incidence between remimazolam and the control group is consistent with recent evidence (Suga et al., 2025; Arias et al., 2025). Moreover, subgroup analysis of delirium subtypes (hyperactive, hypoactive, and mixed) further confirmed the absence of any differences, indicating that remimazolam has no selective effect on the phenotypic presentation of POD (Cai et al., 2025; Yang et al., 2023; Zhang et al., 2026). The duration of delirium, measured in two studies, also showed no statistically significant difference, reinforcing that remimazolam does not alter the clinical course of POD once it develops (Yang et al., 2023; Zhang et al., 2026). Several factors may explain the lack of POD reduction. First, all included studies enrolled elderly patients (mean age 65.2–77.5 years), a population with pre-existing cerebral vulnerability (Duran et al., 2024; Cresto et al., 2025), including age-related neuroinflammation (Mou et al., 2022) and reduced cholinergic activity (Rich et al., 2022), which may override any potential anti-delirium effects of remimazolam. Second, most control groups used dexmedetomidine, an agent with established or proposed anti-delirium properties, which creates a high bar for remimazolam to demonstrate superiority (Liao et al., 2023; Chen et al., 2025). Third, POD assessment tools varied across studies (3D-CAM, CAM, and CAM-ICU) (Cai et al., 2025; Chen et al., 2025; Duan et al., 2024; Liu et al., 2025a; Wang et al., 2025c; Yang et al., 2023; Zhang et al., 2026), introducing minor heterogeneity, though sensitivity analysis confirmed low heterogeneity for POD outcomes, ruling this factor out as a major confounder (Kim et al., 2023). Furthermore, most included studies utilized bispectral index for monitoring the intraoperative anesthesia depth (Chen et al., 2025; Liu et al., 2025a; Yang et al., 2023; Yaqiu et al., 2025; Zhang et al., 2026), a strategy that has been proven to be associated with reduced POD incidence in a prior study (Evered et al., 2021). Notably, a single study showed a trend toward lower POD and DNR at day 7 with remimazolam (Duan et al., 2024), but this was not sustained in the pooled analysis, likely due to small sample size and variability in surgical types.

However, remimazolam was associated with a statistically significant reduction in POCD risk on days 1, 3, or 5, with low heterogeneity and robust sensitivity analysis. POCD, a more persistent cognitive deficit involving memory and executive function, is thought to stem from cerebral microvascular injury, neuroinflammation, and impaired synaptic plasticity (Bhushan et al., 2022; Bowden et al., 2011; Holmgaard et al., 2019). The neuroprotective effect of remimazolam may be attributed to its unique pharmacokinetic and pharmacodynamic properties; as an ultra-short-acting benzodiazepine, it has a rapid offset, reducing the duration of central nervous system depression and minimizing residual sedation that may exacerbate cognitive impairment (Cai et al., 2025; Vellinga et al., 2025; Murrell and Harirforoosh, 2025). Furthermore, remimazolam causes less hemodynamic disturbance than propofol or midazolam (Cai et al., 2025; Wu et al., 2024), preserving cerebral perfusion, which a critical factor in preventing POCD. While the current study did not measure inflammatory markers, one included study reported that remimazolam reduced the incidence of POCD probably due to the reduction of the inflammatory response (Liao et al., 2023). Nevertheless, the POCD analyses are based on a very limited number of small RCTs (Liao et al., 2023; Liu et al., 2025a; Wu et al., 2024). While the pooled estimates show statistical significance with low heterogeneity, the small number of contributing studies means that these findings are preliminary and may be influenced by publication bias or chance. The apparent consistency across time-points (days 1, 3, or 5) is suggestive but does not establish definitive neuroprotection. Additionally, remimazolam also lowers the intraoperative use of remifentanil, which may contribute to POCD reduction, as high opioid doses are associated with increased neuroinflammation and cognitive impairment (Liao et al., 2023; Beloeil et al., 2021). However, there is no statistically significant difference in sufentanil consumption. This finding may be interpreted cautiously for several reasons. First, high statistical heterogeneity indicates variability in effect magnitude across studies. Second, opioid administration was not standardized between groups in the studies, introducing potential confounding by anesthetic protocol. Third, various surgery types may contribute to the different clinical significance. There are no differences in postoperative MMSE scores at 24 h, 72 h, or on day 5, but the remimazolam group had higher scores on day 7 (P, 0.02), indicating delayed but sustained cognitive benefits. The MMSE day 7 score should be interpreted with caution. Extreme heterogeneity (I^2^, 94%) indicates that the pooled estimate does not represent a consistent biological effect.

Remimazolam demonstrated safety advantages, with reduced risk of post-induction hypotension (Duan et al., 2024; Hu et al., 2025; Kuang et al., 2023; Liu et al., 2025a; Wang et al., 2025c; Wu et al., 2024; Yang et al., 2023; Yaqiu et al., 2025), intraoperative bradycardia (Duan et al., 2024; Liu et al., 2025a; Wang et al., 2025c), and postoperative respiratory depression (Wu et al., 2024) compared with that in the controls. These findings are consistent with the pharmacology of remimazolam, which has minimal effects on the cardiovascular and respiratory system, as it does not inhibit the sympathetic nervous system or respiratory center to the same extent as propofol or midazolam (Cai et al., 2025; Wu et al., 2024). Moreover, studies revealed that remimazolam was non-inferior to dexmedetomidine in preventing POD in elderly patients (Chen et al., 2025); it has significant advantages in hemodynamic stability, facilitates faster recovery, and reduces injection pain, establishing it as a preferred anesthetic option for geriatric patients (Hu et al., 2025). Hemodynamic stability is particularly important in elderly patients who have reduced cardiovascular reserve and are at higher risk of cerebral hypoperfusion and subsequent PND (Hu et al., 2025). The lower incidence of respiratory depression also reduces the risk of hypoxemia, a major trigger for neurocognitive impairment (Tang et al., 2025).

Notably, no difference was found in PONV and PACU and hospital stay, indicating that remimazolam does not alter these key perioperative outcomes. The absence of PONV benefit is surprising, as some benzodiazepines have antiemetic properties (Zhang et al., 2023), but it may be due to the use of antiemetic prophylaxis in most studies masking any potential effect of remimazolam. Moreover, the observed neuroprotective effects of remimazolam are critically dependent on the comparator selected. The subgroup analyses of the current study reveal that remimazolam’s neuroprotective effect was not statistically significant difference when compared with propofol. This finding may be attributed to the shared GABAergic mechanism and the well-documented neuroprotective properties of propofol, including its anti-inflammatory and free radical-scavenging effects. Accordingly, the beneficial effect or remimazolam on POCD appears most pronounced when compared with saline or placebo controls, whereas its advantage diminishes when pitted against propofol. These findings underscore the importance of contextualizing remimazolam’s neuroprotective efficacy relative to the pharmacological profile of the comparator agent.

## 5 Limitations of current study

Although the current study has comprehensively analyzed the impact of remimazolam on PND in patients undergoing surgery, several limitations need to be addressed. First, all included studies were conducted in China, limiting the generalizability of findings to other ethnic populations, which may have different pharmacokinetic profiles of remimazolam. Second, some secondary outcomes show high heterogeneity (e.g., MMSE day 7, I^2^, 94%; remifentanil consumption, I^2^, 89%), which may be attributed to the variability in remimazolam dosing (0.1 mg/kg/h–1.5 mg/kg/h), control agents (propofol, dexmedetomidine, midazolam, and saline), surgical types (orthopedic, abdominal, and lung), and anesthesia methods (general vs. spinal). Third, most of the included studies had small sample sizes, increasing the risk of type II error, and only two studies were registered on ClinicalTrials.gov, raising concerns about publication bias. Fourth, the absence of younger adult populations in the included trials limits generalizability to patients under 60 years, and the neurocognitive effects in younger adults remain understudied. Fifth, a major limitation of the current evidence is that PND/POD/POCD was assessed using different cognitive tools, which may have contributed to the heterogeneity observed in the primary outcome. Finally, the long-term cognitive outcomes (beyond 7 days) were not reported, precluding an evaluation of remimazolam’s effect on persistent PND.

## 6 Conclusion

In this systematic review and meta-analysis, remimazolam was not associated with reduced incidence of POD at any assessed time point up to 3 days postoperatively. Remimazolam may be associated with reduced incidence of POD at days 7, and POCD at postoperative days 1,3, and 5, or this finding is based on a limited number of small trials should be interpreted with cautious. Remimazolam may be associated with reduced incidence of early POCD measured by MMSE on postoperative days 1, 3, and 5, but this finding is based on a limited number of small trials should be interpreted with caution. Remimazolam also appears hemodynamically favorable than control sedatives, with reduced post-induction hypotension and intraoperative bradycardia. An opioid-sparing effect was observed for remifentanil but not for sufentanil. However, the certainty of these findings was limited by the small number of contributing studies, short follow-up periods, variable cognitive assessment methods, and the absence of comprehensive neuropsychological batteries. These findings should be considered hypothesis-generating rather than definitive evidence of neuroprotection, and they require confirmatory multicenter trials with standardized neurocognitive assessment and longer follow-up.

## Data Availability

The original contributions presented in the study are included in the article/Supplementary Material; further inquiries can be directed to the corresponding author.
