# Supplementary material for: The impact of remimazolam on postoperative neurocognitive disorder in elderly surgical patients: a systematic review and meta-analysis
Source: Front Pharmacol. 2026 Aug 24;17:1871492. doi: 10.3389/fphar.2026.1871492 (PMC13546993; doi:10.3389/fphar.2026.1871492)
Supplement: Supplementary file 1 [file Supplementaryfile3.doc]

Pubmed, 2026-2-5,74

**#**1 **(((((((((("Postoperative Cognitive Complications"[Mesh]) OR (Postoperative Cognitive Complication[Title/Abstract])) OR (Postoperative Cognitive Decline[Title/Abstract])) OR (Postoperative cognitive dysfunction[Title/Abstract])) OR (Postoperative delirium[Title/Abstract])) OR (Perioperative neurocognitive disorder[Title/Abstract])) OR (Postoperative neurocognitive disorder[Title/Abstract])) OR (Postoperative Cognitive Disorders[Title/Abstract])) OR (Postoperative Cognitive Disorder[Title/Abstract])) OR (Postoperative Dementia[Title/Abstract])) OR (Postoperative Dementias[Title/Abstract])**

**#**2 **Remimazolam**

**#**3 #1 AND #2

WOS, 2026-2-5,117

#1 ((((((((((TS=(Postoperative Cognitive Complications)) OR TS=(Postoperative Cognitive Complication)) OR TS=(Postoperative Cognitive Decline)) OR TS=(postoperative cognitive dysfunction)) OR TS=(postoperative delirium)) OR TS=(perioperative neurocognitive disorder)) OR TS=(postoperative neurocognitive disorder)) OR TS=(Postoperative Cognitive Disorders)) OR TS=(Postoperative Cognitive Disorder)) OR TS=(Postoperative Dementia)) OR TS=(Postoperative Dementias)

#2 TS=(remimazolam)

#3#1 AND #2

#1 'postoperative cognitive dysfunction' OR 'postoperative cognitive complication' OR 'postoperative cognitive decline' OR 'postoperative cognitive dysfunction' OR 'postoperative delirium' OR 'perioperative neurocognitive disorder' OR 'postoperative neurocognitive disorder' OR 'postoperative cognitive disorders' OR 'postoperative cognitive disorder' OR 'postoperative dementia' OR 'postoperative dementias'

#2 'remimazolam'

#31 AND #2

Embase, 2026-2-5,116

Cochrane library, 2026-2-5,319

#1 (postoperative cognitive dysfunction):ti,ab,kw OR (postoperative cognitive complication):ti,ab,kw OR (postoperative cognitive decline):ti,ab,kw OR (postoperative cognitive dysfunction):ti,ab,kw OR (postoperative delirium):ti,ab,kw OR (perioperative neurocognitive disorder):ti,ab,kw OR (postoperative neurocognitive disorder):ti,ab,kw OR (postoperative cognitive disorders):ti,ab,kw OR (postoperative cognitive disorder):ti,ab,kw OR (postoperative cognitive disorder):ti,ab,kw OR (postoperative dementia):ti,ab,kw OR (postoperative dementias):ti,ab,kw

#2 (remimazolam):ti,ab,kw

#31 AND #2
